# Isolation and expression of terminal flower1 (TFL1) gene in clove (*Syzygium aromaticum* L.)

**DOI:** 10.1186/s13104-025-07581-w

**Published:** 2025-12-01

**Authors:** Ireng Darwati, Tri Joko Santoso, Agus Rachmat, Agus Sutanto, Nurliani Bermawie, Atmitri Sisharmini, Rudi Suryadi, Devi Rusmin, Octivia Trisilawati, R. Vitri Garvita, Muchamad Yusron, Suryani Suryani, Tika Anggraeni

**Affiliations:** 1 Research Center for Estate Crops, National Research and Innovation Agency, Cibinong, 16911 Indonesia; 2 Research Center for Horticulture, National Research and Innovation Agency, Cibinong, 16911 Indonesia; 3 Research Center for Genetic Engineering, National Research and Innovation Agency, Cibinong, 16911 Indonesia; 4 Research Center for Food Crops, National Research and Innovation Agency, Cibinong, 16911 Indonesia; 5 Research Center for Applied Botany, National Research and Innovation Agency, Cibinong, 16911 Indonesia; 6 Directorate of Laboratory Management, Research Facilities, and Science Technology Areas, National Research and Innovation Agency, Cibinong, 16911 Indonesia

**Keywords:** Gene expression, Clove, Phase transition, Flowering

## Abstract

**Objective:**

*Syzygium aromaticum* L. is a tropical tree valued for aromatic buds that occur at the branch tips directly affecting yield. The molecular mechanisms of flowering regulation in clove, especially as the role of the TERMINAL FLOWER 1 (*TFL1*) gene, are poorly understood. Most research has emphasized morphology and environmental factors cues, leaving a significant gap in knowledge for improving productivity through breeding. This study aimed to isolate and characterize the *S. aromaticum TFL1* (*SaTFL1*) gene and analyze its expression across different developmental stages. DNA and RNA were extracted from first young leaves of both vegetative (VG) and generative (GG) phase plants, successfully amplifying and sequencing the gene. Gene expression using Reverse Transcriptase PCR on cDNAs derived from both tissue types through comprehensive analysis, including BLAST and phylogenetic studies.

**Results:**

Our results revealed that the deduced amino acid sequence of the partial *SaTFL1* has high identity (86.70% to 98.11%) and similarity (96.22% to 100%) with TFL1/CEN2 proteins from 15 other plant species. *SaTFL1* expression was only found in vegetative tissues but not in generative tissues. The absence of *SaTFL1* expression in generative tissues suggests its key role in promoting vegetative growth and regulating phase transitions. This study is the first to isolate and analyze *TFL1* expression in clove, highlighting its potential function in flowering regulation and improving clove productivity.

**Supplementary Information:**

The online version contains supplementary material available at 10.1186/s13104-025-07581-w.

## Introduction

Clove (*Syzygium aromaticum* L. Merr. & Perr.), an evergreen tree of the Myrtaceae family native to Indonesia’s Moluccan Islands, holds major importance in the global spice trade and as a key income source for farmers. Indonesia ranks first in clove production worldwide, with 135,178 metric tons of raw materials in 2023, contributing 72.8% of global clove production (https://www.fao.org/faostat/en/#data/QCL).

The aromatic flower buds of cloves are rich in essential oils and bioactive compounds such as eugenol, flavonoids, and alkaloids1. Besides their culinary uses, cloves are used in traditional medicine and pharmaceuticals, with eugenol comprising 70–96% of clove oil [1] exhibiting pharmacological properties such as anti-infective activities (antibacterial, antifungal, antiplasmodial, antiviral, anthelmintic), anti-inflamatory activity, analgesic activity, anti-oxidant activity, anticancer activity, antimutagenic and antigenotoxic activities, and modulatory effects [2].

Despite its economic and medicinal value, clove exhibits biennial bearing, alternating between high (“ON”) and low (“OFF”) yield years [3], resulting in inconsistent flowering, unstable yields, and market fluctuations. This pattern may relate to terminal inflorescence formation, with shoot apical meristems generating leaf and axillary primordia. The length of the vegetative (VG) phase varies due to environmental factors and flowering-time genes, with the reproductive transition involving 2–3 cauline leaf primordia followed by floral meristem formation [4].

Currently, specific research on the mechanisms of clove flowering is very limited, especially from a molecular perspective. Existing research focuses on the chemical composition and biological activities of cloves, such as their antifungal, antioxidant, and anticancer properties, rather than their reproductive processes.

Influenced by genetic, environmental, and physiological factors, terminal inflorescences at branch tips are critical for clove flowering. Flowers develop in terminal or axillary cymes, with terminal buds regulated by hormones such as auxins, gibberellins, and cytokinins [5]. Targeting the *Terminal Flower 1* (*TFL1*) gene offers a strategy to address alternate bearings. *TFL1* represses flowering by interacting with *FLOWERING LOCUS D* (*FD*) to inhibit *APETALA1* (*AP1*) and *LEAFY* (*LFY*), maintaining vegetative growth [6], while LFY antagonizes TFL1 to promote flowering [7]. Modulating *TFL1* could synchronize flowering and enhance agronomic outcomes [8].

Flowering time is crucial for reproductive success and crop yield, particularly in perennials with prolonged VG phases and complex seasonal interactions. *TFL1* plays a key role by repressing meristem identity genes and balancing VG and reproductive phases in opposition to *FLOWERING LOCUS T* (*FT*) [9, 10]. Flower induction depends on internal and external cues regulated by the *FT/TFL1* gene family during flower bud development [11]. While the photoperiodic pathway is conserved across species, species-specific variations occur [12]. *TFL1* integrates signals from photoperiod, vernalization, and temperature, and climate change factors, such as rising temperatures and CO₂ levels, can modify flowering via sugar metabolism or direct *FT/TFL1* regulation [13].

*TFL1* has broad functions across horticultural species, inhibits terminal flower formation in *Cucumis sativus* [14, 15], and delays floral transition and maintains inflorescence indeterminacy in *Pisum sativum* [7, 16]. In *Chrysanthemum* (Jimba), overexpressing *TFL1* delays reproductive transition and alters morphology [17]. In Populus spp., flowering is regulated by two *FT-like* (*FT1* and *FT2*) and two *TFL1-like* (*CEN1* and *CEN2*) genes [18], with *FT1* overexpression inducing early flowering in tissue culture [19]. Understanding the genetic and environmental control of flowering transitions can enhance crop value, reduce production costs, and stabilize supply ^[12,20]^. However, the function of *TFL1* in clove remains poorly understood. Therefore, this study aimed to fill this gap by investigating the role of *TFL1* in flowering regulation in clove.

## Methods

### DNA analysis

Genomic DNA was extracted from young clove leaves using the cetyltrimethylammonium bromide method [21]. The DNA’s quality, concentration, and purity were measured using a UV spectrophotometer (Shimadzu, Japan) at 260 nm and by the A260/280 ratio, respectively. Currently, the *TFL1* sequence from *S. aromaticum* is still not available, therefore the primer pair used to amplify the *TFL1* gene sequence in cloves was designed based on the sequence of *Eucalyptus globulus* (*EgTFL1*; GenBank: HQ385322.1), which belong to Myrtaceae, the same family as *S. aromaticum*. The primers were specific to TFL1 gene, i.e. forward (5’-GGTTATGACAGACCCAGATGT-3’) and reverse (5’-CGAACCTGTGGATACCAATG-3’). PCR amplification was carried out in a 20 µL total volume containing 1 μl of genomic DNA (100 ng), 0.5 μl of each primer (20 μM), and 10 μL of KAPA2G Fast ReadyMix. The PCR conditions were as follows: initial denaturation at 94 °C for 2 min; followed by 35 cycles of 94 °C for 15 s, 50 °C for 15 s, and 72 °C for 15 s; and then a final extension at 72 °C for 5 min. The PCR products were visualized on 1% agarose gels in 1 × TBE buffer stained with 0.5 μg/mL of ethidium bromide.

### Sequencing and phylogenetic analysis

Sequencing analysis was performed on the *SaTFL1* PCR amplification products of approximately 700 bp. The deduced amino acid sequence was then compared with sequence in the National Center for Biotechnology Information (NCBI) GenBank non-redundant database using BLASTp [22]. The similarity analysis among the sequences was performed using SIAS (http://imed.med.ucm.es/cgi-bin/sias_new.cgi?jobid=1758871397). The amino acid sequences of the PCR-derived clones of *S. aromaticum* were aligned against known TFL1 proteins from other plants using Geneious Basic (version 5.6.6) [23] to identify conserved domains of TFL1 and predicted substrate binding sites. Three dimensional protein structures were analyzed using the NCBI iCn3D structure viewer (https://www.ncbi.nlm.nih.gov/Structure/icn3d/) to determine its specific biological function such as active and binding sites. A maximum likelihood phylogenetic tree was constructed using MEGA12 [24], with the bootstrap analysis conducted with 1000 replicates.

### RNA isolation and expression analysis

**Fig. 1:**
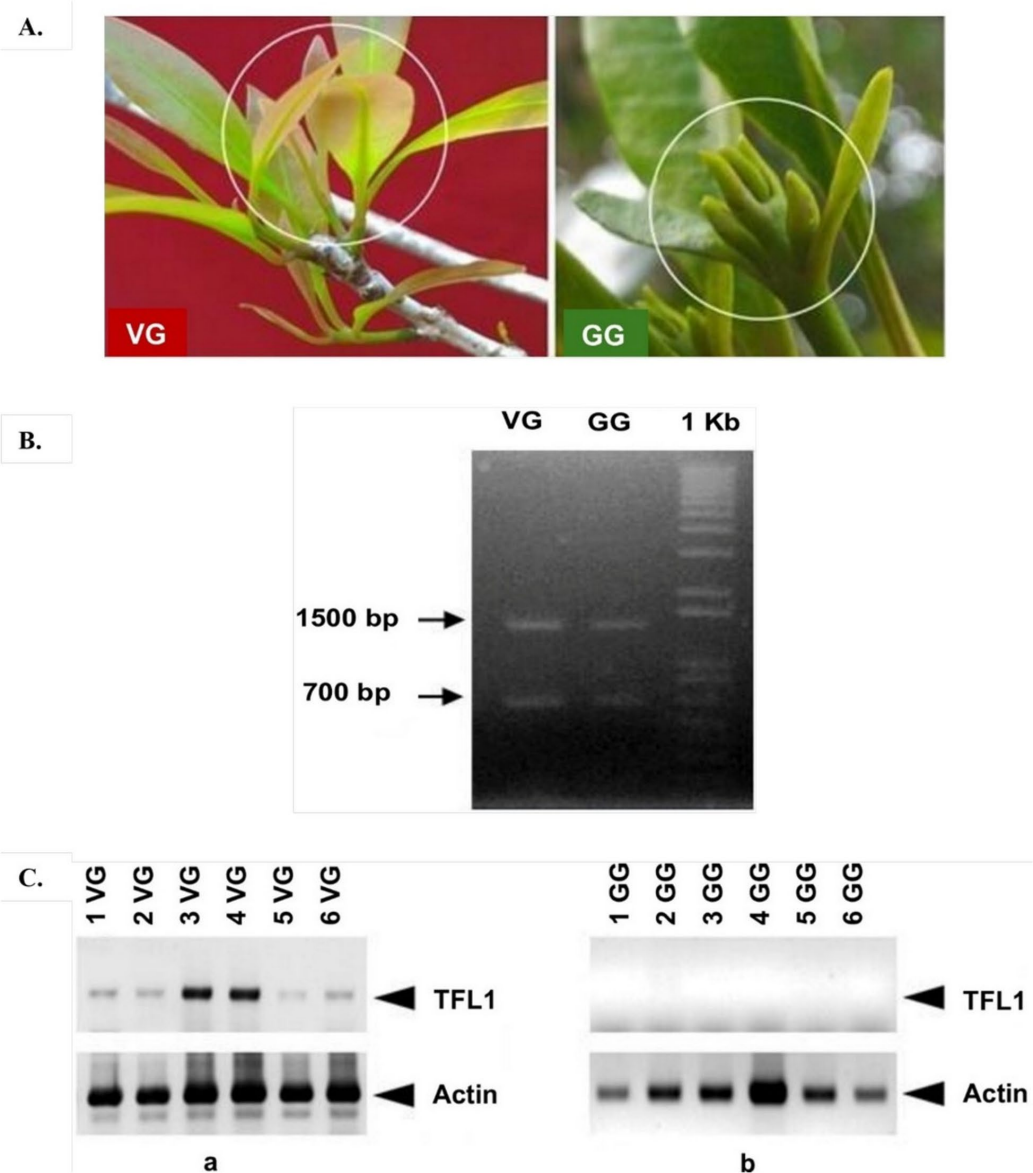
**A** The material obtained from first young leaves during the vegetative (VG) and generative (GG) phases for DNA and RNA extraction. **B** An agarose gel of the PCR products from clove DNA samples using a pair specific TFL1 forward/reverse primers, **C** PCR co-amplification of SaTFL1 sequences was performed using 1 µg cDNA of (**a**) VG and (**b**) GG phases, and actin as a house keeping gene. The amplicon size of the TFL1 gene is 700 bp, and that of actin is 400 bp

Clove leaves (*Syzigium aromaticum* L.) were collected from about 20 years old clove plants grown in the field of the Indonesian Institute for Spices, Medicinal, and Aromatic Plants Engineering and Testing, Bogor with a latitude of 6.576670S and a longitude of 106.785012E. Cultivation procedure followed [25]. Clove leaves were collected from three different plants than bulk at the same developmental stage: before flowering during the vegetative (VG), and at initial flowering time during the generative (GG) phases, corresponding to flower initiation (Fig. 1A).

The expression of *SaTFL* gene expression in the VG and GG phases was analyzed using Reverse Transcriptase-PCR (RT-PCR) technique. The analysis also used actin as the control, which serves as a housekeeping gene. Total RNA was extracted using RNeasy Kits (Qiagen, Germany) according to the manufacturer’s protocol. RNA concentration and quality were assessed spectrophotometrically (NanoDrop 2000; Thermo Fisher Scientific) at 260/280 nm. Next, cDNA was synthesized using the Transcriptor First Strand cDNA Synthesis Kit (Roche). RT-PCR was performed in triplicate on a PCR System (Applied Biosystems, USA)—in 20 μL reactions containing 1 μL of cDNA (0.5 μg), 0.5 μL of each primer (20 μM), and 10 μL of KAPA2G Fast ReadyMix. The amplification conditions were 95 °C for 5 min; followed by 35 cycles of 95 °C for 15 s, 50 °C for 15 s, and 72 °C for 15 s; and then a final extension at 72 °C for 5 min. After the PCR process was complete, the PCR results for both the *SaTFL1* and *actin* genes were visualized on a 1% agarose gel for documentation.

## Results

### Amplification and sequence analysis of *SaTFL1*

**Fig. 2:**
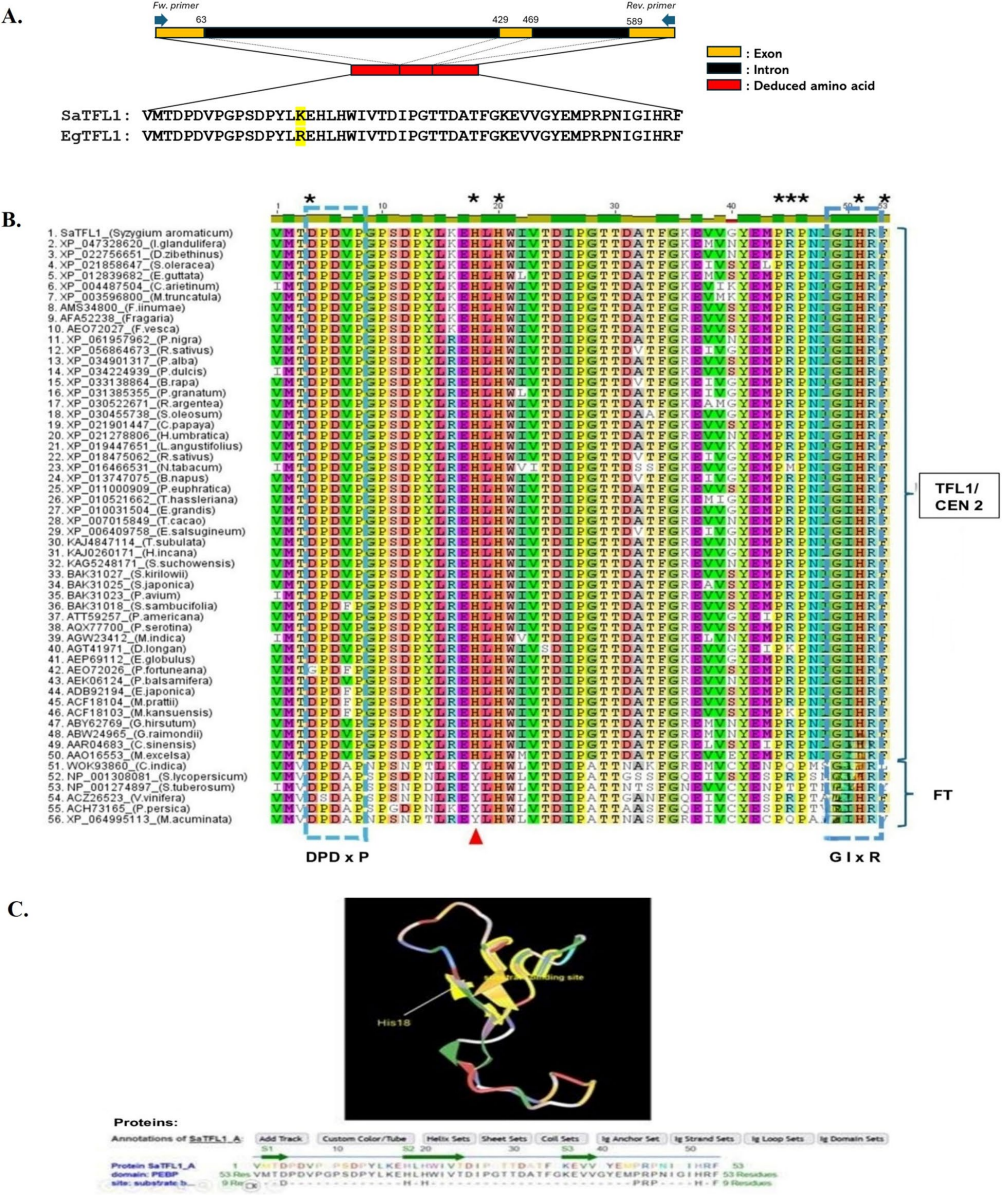
**A** The exon and introns position in the sequenced SaTFL1 fragments and the deduced amino acid sequences of SaTFL1 and EgTFL1. **B** Multiple sequence alignment of the deduced amino acid sequence of SaTFL1, TFL1/CEN2-like proteins from 49 plant species (XP 061957962 [*P. nigra*], XP 056864673 [*R. sativus*], XP 047328620 [*I. glandulifera*], XP 034901317 [*P. alba*], XP 034224939 [*P. dulcis*], XP 033138864 [*B. rapa*], XP 031385355 [*P. granatum*], XP 030522671 [*R. argentea*], XP 030455738 [*S. oleosum*], XP 022756651 [*D. zibethinus*], XP 021901447 [*C. papaya*], XP 021858647 [*S. oleracea*], XP 021278806 [*H. umbratica*], XP 019447651 [*L*. *angustifolius*], XP 018475062 [*R. sativus*], XP 016466531 [*N. tabacum*], XP 013747075 [*B. napus*], XP 012839682 [*E. guttata*], XP 011000909 [*P. euphratica*], XP 010521662 [*T. hassleriana*], XP 010031504 [*E. grandis*], XP 007015849 [*T. cacao*], XP 006409758 [*E. salsugineum*], XP 004487504 [*C. arietinum*], XP 003596800 [*M. truncatula*], KAJ4847114 [*T. subulata*], KAJ0260171 [*H. incana*], KAG5248171 [*S. suchowensis*], BAK31027 [*S. kirilowii*], BAK31025 [*S. japonica*], BAK31023 [*P. avium*], BAK31018 [*S. sambucifolia*], ATT59257 [*P. americana*], AQX77700 [*P. serotina*], AMS34800 [*F. iinumae*], AGT41971 [*D. longan*], AFA52238 [*Fragaria*], AEP69112 [*E. globulus*], AEO72027 [*F. vesca*], AEO72026 [*P. fortuneana*], AEK06124 [*P*. *balsamifera*], ADB92194 [*E. japonica*], ACH73165 [*P. persica*], ACF18104 [*M. prattii*], ACF18103 [*M. kansuensis*], ABY62769 [*G. hirsutum*], ABW24965 [*G. raimondii*], AAR04683 [*C. sinensis*], AAO16553 [*M. excelsa*], and FT-like proteins from six plant species (NP 001308081 [*S. lycopersicum*], NP 001274897 [*S. tuberosum*], ACZ26523 [*V. vinifera*], XP 064995113 [*M. acuminata*], WOK93860 [*C. indica*], AGW23412 [*M. indica*],) deposited in the GenBank database. Highly conserved regions are indicated by blue dashed rectangular boxes, substrate binding sites are marked by asterisks, and a red arrow indicates the critical residue (His/Tyr) that distinguishes TFL1/CEN- and FT-like proteins. **C** The three dimensional structure of the partial protein sequence of SaTFL1, showing the substrate binding sites (amino acids highlighted in yellow): Asp (4), His (18, 20, and 51), Pro (44, 46, and 53), and Arg (45)

PCR amplification was performed using the amount of DNA on clove DNA samples produced two distinct bands of ~ 650 and ~ 1500 bp (Fig. 1B). The 1500 bp fragment showed no homology to TFL1 (data not shown), while the ~ 650 bp fragment, consisting of two introns measuring 484 bp and three exons measuring 159 bp, encodes 53 amino acids (Fig. 2A). The sequence of these 53 amino acid residues showed homology to a partial sequence of TFL1 (Fig. 2A).

A BLASTp [22] analysis revealed that the deduced amino acid sequence of the PCR product shared 98.11% identity with EgTFL1 (GenBank: AEP69112.1), with an e-value of 1 × 10 − 39 (Table 1). It also identified selected TFL1/CEN2 proteins from various plant species (Table 1) and selected them for further analysis. It showed that the deduced amino acid sequence of PCR product shared 86.70% to 98.11% identity and 96,22% to 100% similarity with 15 other plant TFL1/CEN2 proteins deposited in the GenBank database, with several amino acid substitutions observed (Fig. 2B). The 15 other plants belong to the Dicotyledonae consisted of Myrtaceae (4 species), Rosaceae (4 species), Solanaceae (2 species), Malvaceae (1 species), Passifloraceae (1 species), Salisacea (1 species), Lauraceae (1 species), and Brasicaeae (1 species). For further analysis purposes, the sequence of the PCR product from clove was coded as SaTFL1. A multiple alignment analysis of the deduced amino acid sequence of SaTFL1 with TFL1/CEN2-like proteins of 49 plant species identified conserved domains (D-P-D-V-P and G-I-H-R) and predicted substrate binding sites (Fig. 2B), supporting the classification of SaTFL1 as a TFL1 homolog. The results of the multiple alignment analysis also showed the differences in amino acid residues between TFL1/CEN2-like and six FT-like proteins. This result of multiple alignment analysis was used to generate a phylogenetic tree.

**Table 1:** The result of BLASTp [22] analysis of deduced amino acid sequence homology comparison of SaTFL1 with 15 TFL1/CEN2 proteins of other plants deposited in GenBank

Accession	Description	Family	E-value	Identity (%)	Similarity (%)
AEP69112.1	Terminal flower 1-like protein (*Eucalyptus globulus*)	Myrtaceae	1e−39	98.11	100
XP_010031504.1	PREDICTED: Protein CENTRORADIALIS-like (*Eucalyptus grandis*)	Myrtaceae	2e−39	98.11	100
XP_030455738.1	protein CENTRORADIALIS-like [Syzygium oleosum]	Myrtaceae	7e−39	96.23	98.11
AEO72027.1	TFL1-like protein [*Fragaria vesca*]	Rosaceae	7e−39	96.23	98.11
AAO16553.1	Terminal flower 1-like protein (*Metrosideros excelsa*)	Myrtaceae	2e−38	94.34	96.22
KAJ4847114.1	CEN-like protein 2 [Turnera subulata]	Passifloracea	2e−38	94.34	98.11
KAG5248171.1	TFL1a family protein [Salix suchowensis]	Salisaceae	2e−38	94.34	98.11
ATT59257.1	TERMINAL FLOWER1 [*Persea americana*]	Lauraceae	2e−38	94.34	98.11
ADB92194.1	TFL1-like protein [*Eriobotrya japonica*]	Rosaceae	4e−38	92.45	96.22
BAK31023.1	TFL1-like protein, partial [*Prunus avium*]	Rosaceae	8e−39	92.45	98.11
NP_001275478.1	Terminal flower 1 protein [Solanum tuberosum]	Solanaceae	1e−37	90.57	96.22
ACF18104.1	Terminal flower 1 [Malus prattiil	Rosaceae	4e−38	92.45	96.22
XP_016466531.1	CEN-like protein 2 [Nicotiana tabacum]	Solanaceae	7e−37	86.70	96.22
XP_007015849.1	PREDICTED: CEN-like protein 2 [Theobroma cacao]	Malvaceae	2e−38	94.34	98.11
XP_033138864	Protein CENTRORADIALIS-like [Brassica rapa]	Brasicaceae	1e−38	94.34	98.11

Prediction of the three dimentional structure of the partial amino acid sequence further confirmed that SaTFL1 belongs to the phosphatidylethanolamine-binding protein (PEBP) family (Fig. 2C), revealing eight substrate binding residues, including aspartate (position 4), proline (position 44, 46, and 53), histidine (positions 18, 20, and 51), and arginine (position 45).

**Fig. 3:**
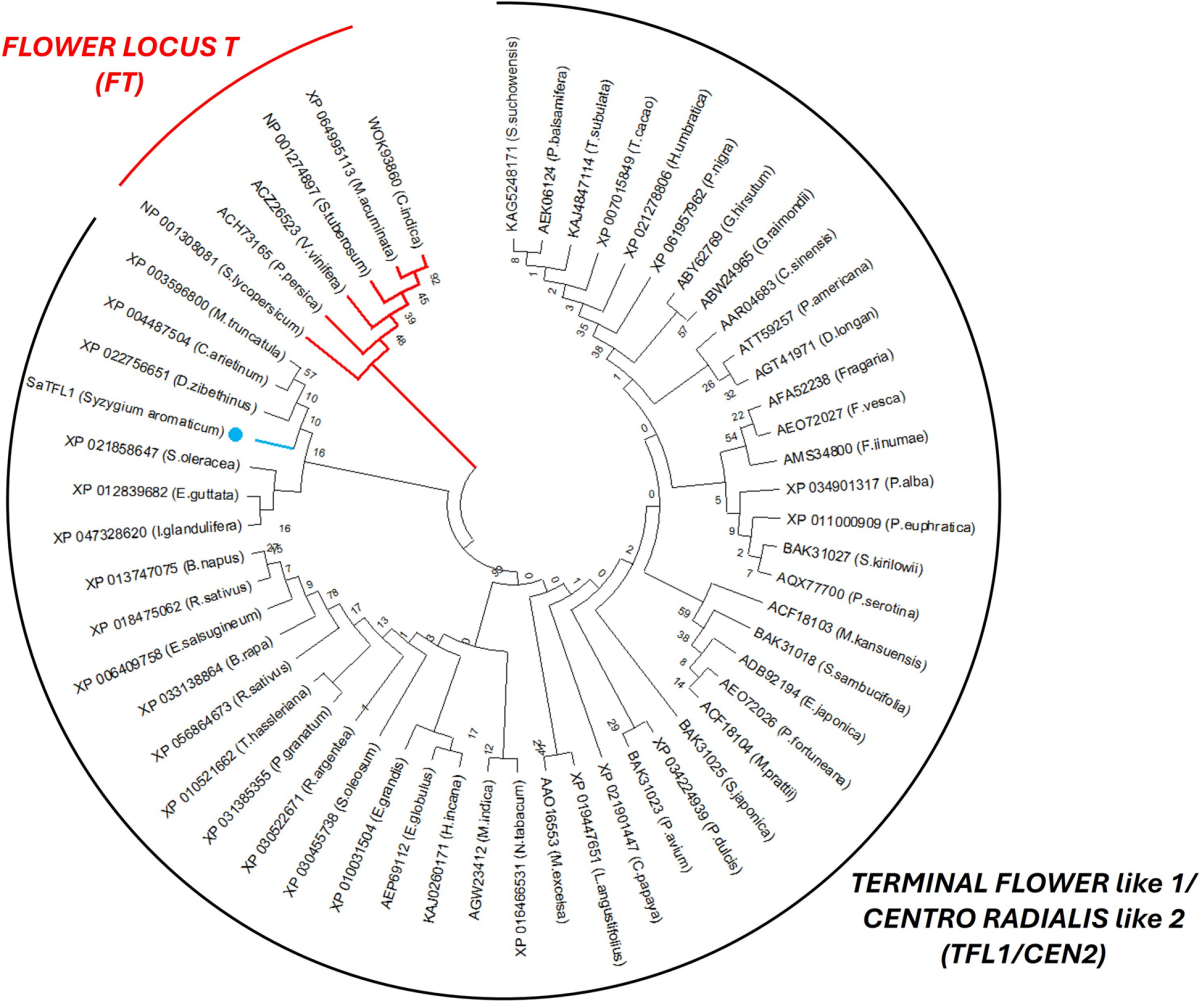
The phylogenetic tree of SaTFL1 (blue marker), TFL1/CEN-like proteins from 49 plant species, and FT-like proteins from six plant species in the GenBank database. Construction of the phylogenetic tree was developed using the maximum likelihood method in MEGA12 software. Bootstrap values (1000 replicates) are shown on the branches. Red branches denote FT-like proteins, and black branches denote TFL1/CEN2-like proteins

Phylogenetic tree of deduced amino acid of SaTFL1 with TFL1/CEN2- and FT-like proteins from 55 plant species deposited in the GenBank database is shown in Fig. 3. Based on phylogenetic analysis, two main branches were revealed, designated as FLOWER LOCUS T (FT) and TERMINAL FLOWER locus like 1 / CENTRO RADIALIS like 2 (TFL1/CEN2). The TFL1/CEN2 cluster is divided into two subclusters. SaTFL1 is in one subcluster with *M. truncata*, *C. arietinum*, *D. zibethinus*, *S. oleracea*, *E. guttata* and *I. glandulifera*. While TFL1 from *Eucalyptus globulus* is in another subcluster. This is due to the differences in evolution pattern and sequence character of each taxon [26].

### SaTFL1 expression analysis

The results of *SaTFL1* gene expression analysis using RT-PCR showed the presence of cDNA of *SaTFL1* in clove leaf tissue during the early vegetative phase (VG), but it was undetectable during the early generative (GG) phase (Fig. 1C).

## Discussion

In this study, we successfully isolated and characterized a partial sequence of the *SaTFL1* gene in clove and analyzed its expression in leaves during the VG and GG phases. The primers were designed based on *TFL1* gene alignments dicotyledonous plants, indicating successful amplification of a *TFL1* homolog in clove. The PCR products were sequenced to confirm the amplified fragment’s identity. The 645 bp sequence showed homology to the *TFL1* gene from *Eucalyptus globulus* (*EgTFL1*; GenBank: HQ385322.1), a member of the same family (Myrtaceace) as clove.

BLASTp analysis confirmed that the deduced amino acid sequence of the PCR product originated from clove was homologous to the TFL1 or CEN2-like protein, as shown in Table 1. Furthermore, multiple sequence alignment revealed that the deduced amino acid sequence of the PCR product contained conserved amino acid regions and substrate binding sites characteristic of the phosphatidylethanolamine-binding protein (PEBP) family, including TFL1/CEN2-like and FT-like proteins. Its predicted three dimensional structure (Fig. 2C) identified eight substrate-binding sites that are likely to serve as functional active sites in the protein.

A key distinction between TFL1/CEN2- and FT-like- proteins is amino acid residue (H/Y) at position 18 (Fig. 2B), a substitution critical for functional divergence [27]. Functional studies in *Arabidopsis thaliana* have demonstrated that 35::FT (Y85H) mutants failed to induce terminal flower formation, similar to 35::TFL1 mutants, highlighting the importance of this residue [10].

To elucidate the evolutionary relationship of SaTFL1, a phylogenetic analysis was conducted using TFL1/CEN2- and FT-like proteins from 55 plant species deposited in the GenBank database. Phylogenetic analysis revealed that although SaTFL1 shares a high sequence identity with TFL1 from *Eucalyptus globulus* (98.11%), it located in a different subcluster of the phylogenetic tree, clustering more closely with *Durio zibethinus*. This suggests that distinct evolutionary patterns and sequence characteristics are present among the analyzed taxa. The analysis highlights the functional importance of TFL genes in regulating the timing of the vegetative phase transition and in maintaining indeterminate meristems during plant development.

*SaTFL1* expression levels varied among individual leaves in the VG phase. In clove, flowering occurs at the terminal end of branches, and optimal flower production depends on the number of flowering-capable branches. Flowering transition and branch architecture are regulated by a network of genes, many of which have been well-characterized in various plant species [10, 17, 28]. However, their role in clove remains unexplored.

The transition from the VG to the GG phase is regulated by members of the PEBP family. In *A. thaliana*, the PEBP family includes *FT, TWIN SISTER OF FT* (*TSF*), *TFL1*, *BROTHER OF FT AND TFL1* (*BFT*), *CENTRORADIALI* (*ATC*), and *MOTHER OF FT AND TFL1* (*MFT*). Among these, *FT*, *TSF*, and *MFT* promote flowering, whereas *TFL1*, *BFT*, and *ATC* repress floral identity. Despite sharing 54–61% sequence identity, *FT* and *TFL1* exhibit antagonistic functions and have received attention [29].

TFL1 proteins are known to translocate from the leaves to the plant meristem, influencing floral development. Consistently, our expression analysis revealed that *SaTFL1* transcripts were detectable in leaves during the VG phase but not the GG phase (Fig. 1C), suggesting *SaTFL1* is downregulated upon floral transition. In citrus plants, the expression pattern of *TFL* transcripts differed from that observed in other species. No transcripts were detected in vegetative tissues such as leaves, stems, and roots Instead, *TFL* transcripts were present in all organs of fully developed flowers [30]. The functional significance of this floral-specific expression remains unclear, further studies are required to elucidate its role.

This interpretation remains provisional, as it has not yet been validated using quantitative RT-PCR. In this study, however, the presence of SaTFL1 cDNA at the VG stage was confirmed. The observed variation in SaTFL1 transcript levels among VG-phase leaf samples (Fig. 1C) may reflect differences in leaf developmental stage or sampling position. These factors should be carefully considered in future expression studies. Further research including analyzing of *FT* and other flowering regulators such as *TSF* and *MFT*, is needed to clarify the role of *SaTFL1* in the VG-to-GG transition.

Understanding the regulation of vegetative shoot formation and flowering in perennial crops such as clove is crucial for optimizing productivity. The successful isolation of *SaTFL1* provides a foundation for dissecting the genetic control of clove architecture and flowering. Future research should focus on elucidating the interaction between *SaTFL1* and flower-promoting genes, which may offer strategies for enhancing clove flower production through genetic modification.

Analysis across a wide range of distantly related species have demonstrated that *TFL-like* genes play a crucial role in plant development, primarily by regulating the timing of the vegetative phase transition and by maintaining indeterminate meristems. However, the specific molecular mechanisms underlaying phase change in perennial tree crops remain largely uncharacterized. In *Citrus sinensis*, elevated expression levels of a functional homologue have been shown to correlate strongly with the juvenile phase Whether targeted downregulation of *CsTFL* expression through transgenic approaches could shorten this prolonged juvenile period in citru and other perennial crops of agronomic importance such as clove remains to be investigated.

### Limitations

- Information regarding genes related to flowering in clove plants is still very limited.
- The results of this research are the basis for revealing the flowering mechanism in clove plants and can be used to improve clove plant management.

## Supplementary Information

Supplementary material 1.

Supplementary material 2.

## Data Availability

The sequence data of SaTFL1 has been deposited in NCBI with the accession no. PV911194 can be accessed at. https://www.ncbi.nlm.nih.gov/nuccore/PV911194. The datasets of Fig. 2 and Fig. 3 generated and/or analyzed during the current study are available from the corresponding author on reasonable request.

## Abbreviations

*TFL1*: TERMINAL FLOWER 1
*SaTFL1*: *S. aromaticum TFL1*
VG: Vegetative
GG: Generative
*FD*: *FLOWERING LOCU* *S* *D*
*AP1*: *APETALA1*
*LFY*: *LEAFY*
*FT*: *FLOWERING LOCU* *S* *T*
TSF: TWIN SISTER OF FT
BFT: BROTHER OF FT AND TFL1
ATC: CENTRORADIALI
MFT: MOTHER OF FT AND TFL1
DNA: Deoxyribonucleic acid
RNA: Ribonucleic acid
PCR: Polymerase chain reaction
RT-PCR: Reverse transcription polymerase chain reaction
cDNA: Copy DNA
