# Supplementary material for: Isolation and expression of terminal flower1 (TFL1) gene in clove (Syzygium aromaticum L.)
Source: BMC Res Notes. 2025 Dec 1;19:4. doi: 10.1186/s13104-025-07581-w (PMC12776960; doi:10.1186/s13104-025-07581-w)

Analysis of TFL1 gene expression in cloves

a. Analysis of *TFL1* gene expression in young leaves from the first node of generative stage

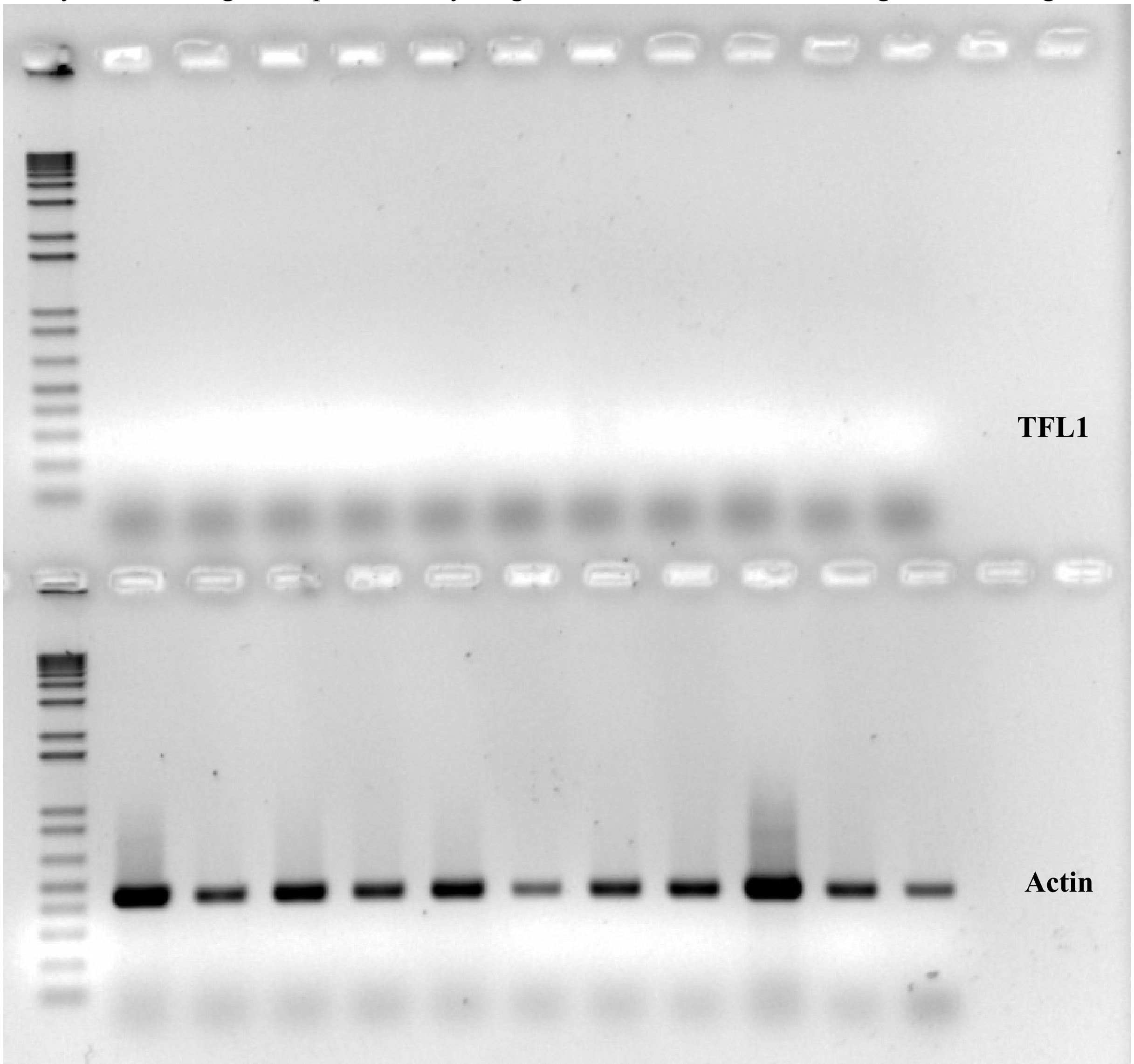

b. Analysis of *TFL1* gene expression in young leaves from the first node of vegetative stage

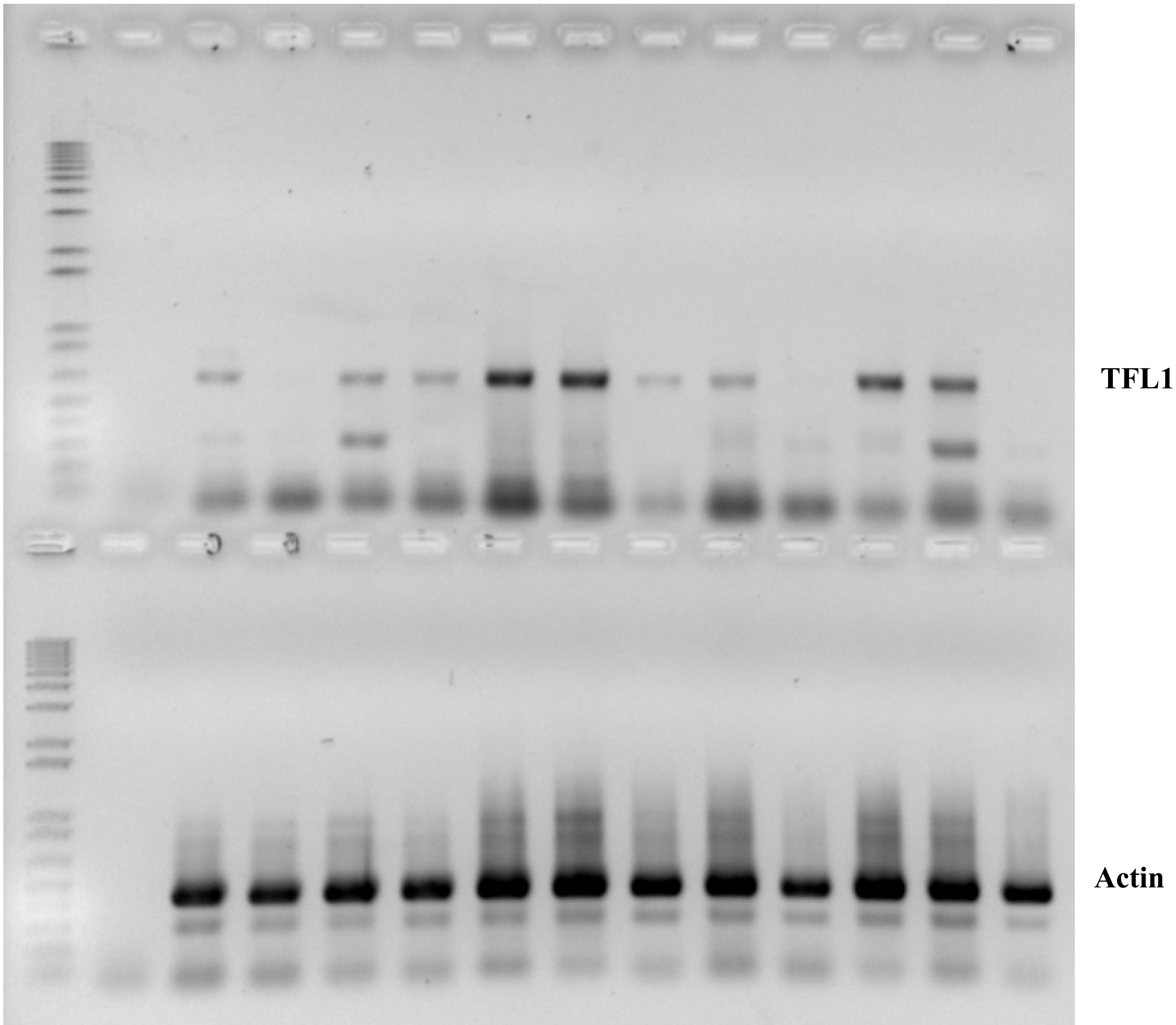

Supplement: Supplementary file 1 — Supplementary material 1. [file 13104_2025_7581_MOESM1_ESM.pdf]
