# Supplementary material for: Isolation and expression of terminal flower1 (TFL1) gene in clove (Syzygium aromaticum L.)
Source: BMC Res Notes. 2025 Dec 1;19:4. doi: 10.1186/s13104-025-07581-w (PMC12776960; doi:10.1186/s13104-025-07581-w)
Supplement: Supplementary file 2 — Supplementary material 2. [file 13104_2025_7581_MOESM2_ESM.pdf]

Gel electrophoresis of TFL1 from leaves samples of Clove

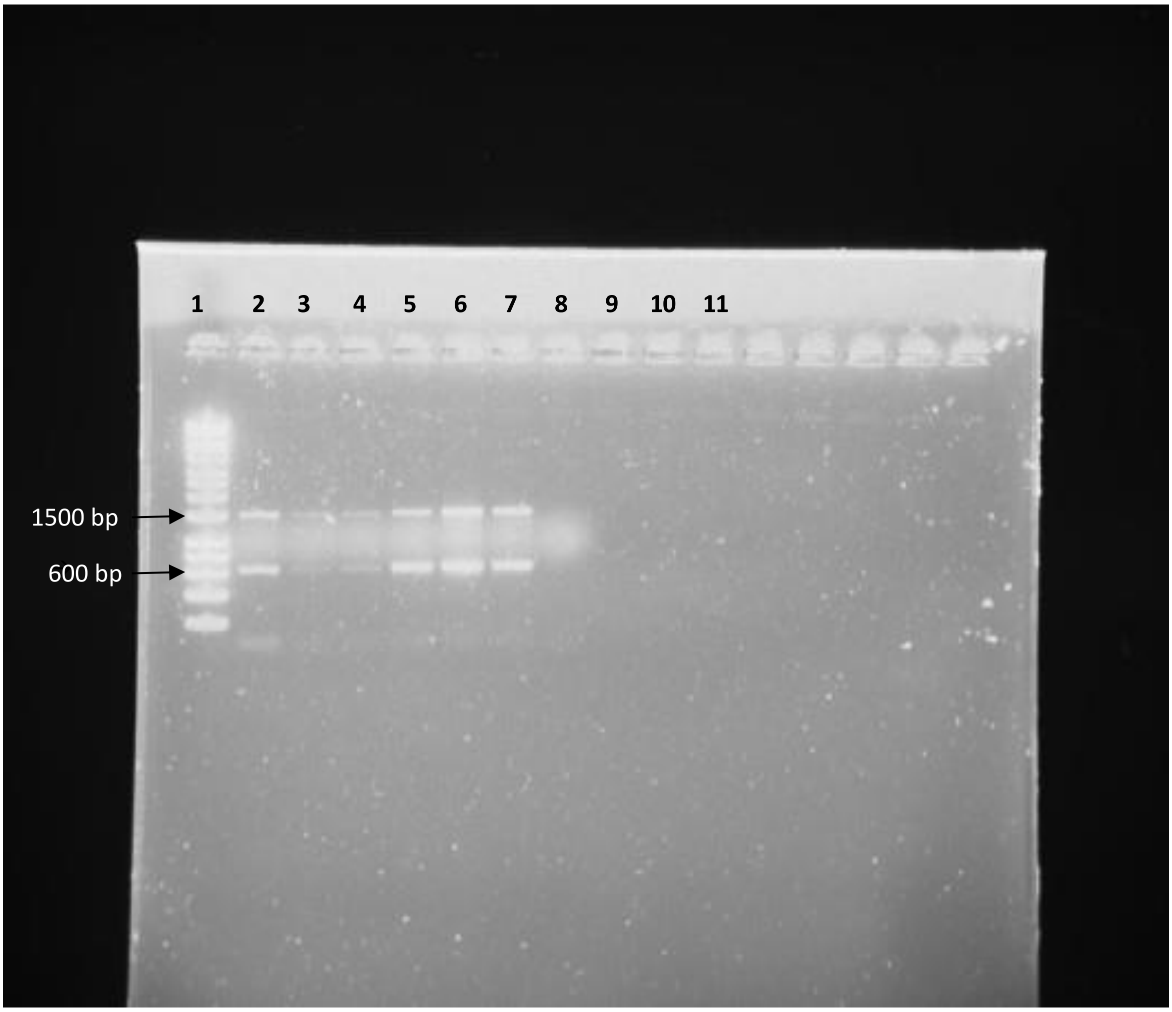

Line 1: Ladder
Line 2: Sample 1, 40 mg
Line 3: Sample 1, 50 mg
Line 4: Sample 1, 60 mg
Line 5: sample 2, 40 mg
Line 6: Sample 2, 50 mg
Line 7 : Sample 2, 60 mg
Line 8: Sample 3, 40 mg
Line 9: Sample 3, 50 mg
Line 10 : Sample 3, 60 mg
Line 11: Sample 3, 40 mg
Sample 1: Leaf from first internode
Sampel 2: Leaf from second internode
Sampel 3: Leaf from third internode
